# Prevalence and predictors of unknown HIV status among women delivering in Mulago National Referral Hospital, Kampala, Uganda

**DOI:** 10.4314/ahs.v17i4.3

**Published:** 2017-12

**Authors:** Emily C Namara-Lugolobi, Gertrude Nakigozi, Zikulah Namukwaya, Dan K Kaye, Edith Nakku-Joloba

**Affiliations:** 1 Makerere University — Johns Hopkins University (MU-JHU) Research Collaboration, Kampala, Uganda; 2 Makerere University College of Health Sciences, School of Public Health, Kampala, Uganda; 3 Makerere University College of Health Sciences, Department of Obstetrics and Gynecology, Kampala, Uganda

**Keywords:** Unknown HIV status, women delivering, Mulago National Referral Hospital, Kampala, Uganda

## Abstract

**Introduction:**

Knowledge of a person's HIV status during pregnancy is critical for prevention of mother to child transmission of HIV.

**Objectives:**

To determine the prevalence and factors associated with unknown HIV status among women delivering in Mulago Hospital.

**Methods:**

This was a cross-sectional study of women that had just delivered. The women's demographic characteristics, health seeking behaviour, health system-related factors and knowledge on PMTCT were collected. Fisher's exact test, Wilcoxon rank sum test and logistic regression were used to test associations.

**Results:**

The prevalence of unknown HIV status was 2.6% (10/382). Attending ANC at higher level facilities (OR =0.1 95% CI 0.0 – 0.4) and having been counselled for HIV testing during ANC (OR=0.1, 95% CI 0.0 – 0.4) were associated with likelihood of having a known HIV status. Out of the ten women with unknown HIV status, 4/6 who attended ANC in public/government accredited health facilities “opted out” of HIV testing due to personal reasons. Among the four who attended ANC in private clinics, two were not offered HIV testing and one “opted out”.

**Conclusion:**

Most participants had a known HIV status at labour (97%). Private clinics need to be supported to provide free quality HCT services in ANC.

## Introduction

Mother to Child transmission (MTCT) of HIV accounts for more than 90% of all new paediatric HIV infections and occurs in utero, during labour and delivery and during breastfeeding^1^. Without any intervention, the MTCT rate ranges from 15% to 45%^2^. The use of anti-retroviral therapy (ART) and elective caesarean section has reduced MTCT rates to less than 2% in non-breastfeeding populations^2^. Even in breastfeeding populations, studies have demonstrated that timely anti-retroviral drugs can reduce mother to child transmission of HIV to 5% or less^3^–^5^.

Use of Provider Initiated Counselling and Testing (PICT) has been associated with increased uptake of HIV testing in health facilities^6^. However, in sub-Saharan Africa, uptake of HIV Counselling and Testing (HCT) during pregnancy has not been optimum. For example, in 2011, the UNAIDS estimated that only 61% of all pregnant women in sub Saharan Africa received HIV testing. In accordance with the WHO recommendations^6^, the Uganda Ministry of Health changed policy from Voluntary Counselling and Testing to PICT in 2005^7^. In spite of the policy change, only 66.4% of pregnant women were tested in Uganda in 2011^8^. This proportion has tremendously improved from 21% in 2006^9^ but it was still lower than the coverage target of 80% by 2010 that was set at the United Nations General Assembly Special Session on HIV AIDS (UNGASS)^10^,^11^.

Previous studies have shown that the fear of stigma and discrimination, service delivery factors, knowledge on Prevention of Mother to Child Transmission of HIV (PMTCT), poverty and individual health seeking behaviors affect the uptake of HCT during pregnancy^12^–^14^. The objective of this study was to determine the prevalence and factors associated with unknown HIV status among the women delivering in Mulago Hospital after introduction of PICT in Uganda.

## Methods

This was a cross-sectional study on women who had just delivered and were admitted on the post-natal ward in Mulago Hospital.

### Study site and setting

Mulago National referral Hospital has three labour wards; upper Mulago, Ward 6D and E and ward 5C (lower Mulago). The labour ward in upper Mulago handles low risk pregnant women. Labour ward 6D & E attends to private patients who must have been booked by an obstetrician. The labour ward on 5C admits high risk women and referrals from the formal and informal health sector. There are about 2300 deliveries at ward 5C per month. The HIV status of every woman is identified at admission, those with unknown HIV status receive HCT and if positive, are given emergency ARVs for PMTCT. The study site was the post-natal ward.

### Study population/eligibility Criteria/Selection Procedure

Women who had delivered and were admitted on the post-natal ward were approached by the research assistants including those aged less than 18 years (emancipated minors). Those who consented to participate in the study were enrolled. Women with life threatening conditions (for example post-partum haemorrhage, eclampsia and unconscious patients) and the mentally impaired were excluded.

### Sample size calculation:

The sample size was determined using the Leslie Kish Formula for random sampling using proportions (Kish 1965). We assumed the population proportion of women who did not test in Uganda as 66.4% (UNAIDS Progress report 2011). Using 5% as the level of significance, a power of 80%, and an additional 10% to cater for none response, the sample size was calculated as 380 women.

### Data collection

Between 14^th^ March to 30^th^ April 2013, data was collected by research assistants. The women who had delivered and admitted on Ward 5B and C were approached consecutively and asked to participate in the study. Those who were eligible were asked to give consent. Interviewer-administered questionnaires collected information on the participants' socio-demographic characteristics. To assess health seeking behaviour, participants were asked whether they attended ANC, level of health facility, number of times they attended and acceptance to test for HIV.

Having an unknown HIV status at presentation to the labour ward was determined by asking participants' knowledge of their HIV status. Those with unknown HIV status were asked to explain how they missed testing during pregnancy. In order to estimate accessibility of health facilities, women were asked about the distance and transport costs to health facilities. Other service delivery factors assessed included, HCT services and referral if HCT was not offered. The questions to determine mother's knowledge on PMTCT included knowledge of the importance of HIV testing in pregnancy, how MTCT of HIV occurs and available PMTCT interventions. Completed questionnaires were edited then entered into Epi info by a data assistant.

### Quality control

The research assistants were trained to use the data collection tools, the data collection instruments were pre-tested and the data collected was reviewed by the investigator and errors corrected in real time before data entry.

### Ethical review

The protocol was reviewed and approved by the Makerere University School of Public Health Higher Degrees Research and Ethics and Committee (HDREC), and Mulago Hospital Research and Ethics Committee(protocol MREC:348). Written informed consent was obtained from all study participants. Data was accessed only by authorized personnel. Identification of participants was by unique numbers and data was stored in password protected programs.

### Statistical analysis

The outcome variable “having an unknown HIV status at presentation to the labour ward” was determined by asking participants' knowledge of their HIV status. The independent variables included; demographic characteristics, service delivery factors, health seeking behaviour and knowledge on PMTCT. Statistical analyses used Stata Version 12 (Stata Corporation, 4905 Lakeway Drive, College Station, TX 77845, USA). Proportions of women with unknown HIV status were estimated. In bivariate analysis non-parametric methods (Wilcoxon runk sum test for continuous variables and Fishers exact test for categorical variables) were used to assess associations. Factors with p values less than 0.2 in bivariate analysis and with theoretical relevance to the outcome were considered for logistic regression, both for unadjusted and adjusted analysis. Only factors with p values less than 0.05 on multivariate analysis were considered statistically significant.

## Results

Participants' median age was 24 years with the interquartile range of 21 to 28 years. About 40% were delivering for the first time (prime-parous), 47.9% were giving birth for the second to fourth time while only 10% were delivering for more than the fourth time (multi-parous). The majority were married (84%) and 78% of the married women were in monogamous relationships. About 62% had attained an education level of secondary education or higher, while 6.6% had no formal education.

### Unknown HIV status at labour

Analysis of maternal health seeking behaviour showed that the majority had ever been tested for HIV during the current pregnancy and only 10 women (2.6%, 95% CI 1.3% – 4.8%) reported in labour with unknown HIV status. Among those with known status, 5% (20/372) reported without documentation of HIV testing and so were considered as having unknown HIV status by the attending health workers and tested again at labour. The reasons given for not testing during the antenatal period have been summarised in table 1.

**Table 1:** Reasons as to why participants did not test for HIV during the antenatal period

Reason	Number	Health centre attended for ANC
Mother did not want to know her results	3	HC III, HC IV
Had no money to pay for the test	2	Private clinic, HC IV
The nurse did not mention anything about HIV testing	1	Private Clinic
There was no health worker responsible for testing	1	Private clinic
Reluctant to take the test	1	Private clinic
One was counselled and tested but did not receive results because it was late and the nurses were tired	1	HC IV
Fear of needle pricks	1	HC III

### Testing at the labour ward

All the women who presented with unknown HIV status were tested at labour. Two were tested before delivery and eight after delivery. The reasons for testing after delivery included reporting in second stage of labour (4/8), the test was not offered (3/8) and one gave birth before arrival to the hospital.

### Bivariate analysis

Bivariate analysis showed there was no significant demographic factor (p ≤0.05) associated with having an unknown HIV status in labour as shown in table 2.

**Table 2:** Bivariate analysis for demographic characteristics

Variable	*Unknown HIV status* *(N=10)*	*Known HIV* *status* *(N=372)*	*Total* *(N=382)*	*p value*
**Age (years)**	**n (%)**	**n (%)**	**n (%)**	
Less than 20	3(5.1)	56(94.9)	59(100)	0.224
20 – 30	5(1.9)	261(98.1)	266(100)	
Greater than 30	2(3.7)	52(96.3)	54(100)	
**Parity**	**n (%)**	**n (%)**	**n (%)**	
1	4(2.5)	154(97.5)	158(100)	0.725
2 – 4	6(3.3)	174(96.7)	180(100)	
Multi parity	0(0.00)	38(100)	38(100)	
**Religion**	**n (%)**	**n (%)**	**n (%)**	
Born again	0 (0.0)	52 (100)	52 (100)	0.235
Catholic	1 (1.7)	118 (98.3)	120 (100)	
Moslems	4 (4.1)	93 (95.9)	97 (100)	
Protestant	5(4.8)	100 (95.2)	105 (100)	
Others	0 (0.0)	6 (100)	6 (100)	
**Education level**	**n (%)**	**n (%)**	**n (%)**	
None	1 (4.0)	24 (96.0)	25 (100)	0.743
Primary	3 (2.5)	117 (97.5)	120 (100)	
Secondary	6 (2.0)	191 (98.0)	197 (100)	
Tertiary	0 (0.0)	37 (100)	37 (100)	
**Marital status**	**n (%)**	**n (%)**	**n (%)**	
Single	4 (6.8)	55 (93.2)	59 (100)	0.126
Married	6(1.9)	314 (98.1)	320 (100)	
Widowed	0 (0.0)	1 (100)	1 (100)	
Separated/divorced	0 (0.0)	2 (100)	2 (100)	
**Household monthly** **income (UGx)**	**n (%)**	**n (%)**	**n (%)**	
Less than 5000	0 (0.0)	4 (100)	4 (100)	0.460
5000 – 500,000	8 (3.5)	219 (96.5)	227 (100)	
500,001 – 1,000,000	1 (1.8)	107 (98.2)	109 (100)	
Above 1,000,000	0 (0.0)	21 (100)	21 (100)	

### Health Seeking Behavioural factors

Table 3 summarises bivariate analysis for health seeking behaviour. All the participants attended ANC during the just ended pregnancy and 50% had attended in a hospital setting. Participants whose HIV status was known attended their first ANC two weeks earlier than those with unknown HIV status (20 and 22 weeks of gestation) but this was not statistically significant (p value= 0.726, Wilcoxon runk sum test).

**Table 3:** Bivariate analysis for health seeking behavior

	*Unknown HIV* *status(N=10)*	*Known HIV* *status (N=372)*	*Total(N=382)*	*p value*
**Attended ANC during pregnancy**	**n (%)**	**n (%)**	**n (%)**	
Yes	10 (2.6)	372 (97.4)	382 (100)	−------
**Gestational Age at first ANC visit** **Median(IQR)**	22(20–28)	20(16–28)	20(16–28)	0.726
**Level of health center where mother** **attended ANC**	**n (%)**	**n (%)**	**n (%)**	
Private clinic	4(17.4)	19 (82.6)	23 (100)	<0.001
Health center II/ Health Centre III	2(5.7)	33 (94.3)	35 (100)	
Health center IV/ Hospital	4 (1.2)	320 (98.8)	324 (100)	
(Government/NGO)				
**Frequency of ANC attendance**	**n (%)**	**n (%)**	**n (%)**	
Twice or less	4 (36)	78 (64.0)	82 (100)	0.235
More than twice	6 (2.0)	287(98.0)	293 (100)	
**Male partner Support**	**n (%)**	**n(%)**	**n(%)**	
Partner did not attend ANC	9(3.5)	245(96.5)	254(100)	0.176
Attended ANC with Partner	1(0.8)	124(99.2)	125(100)	

There was a significant difference in the level of health facility where mother attended ANC (p value <0.001, Fishers exact test) and in the number of times the mother attended ANC (p value = 0.026, Fisher's exact test) between those presenting with a known and those with unknown HIV status.

Overall, 33% of the participants attended ANC with their partners. There was no difference in partner support between those with known and those with unknown HIV status (p value =0.176, Fisher's exact test).

### Service delivery factors at the Health facility where mother attended ANC

Ninety percent (90%) of the participants with unknown and 95.7% of those with known HIV status had a health facility that offers HIV testing to pregnant women in their locality. The median distance to these facilities was 0.5 kilometres for both categories of women. There was no statistically significant difference between the two groups in the average distance and average transport costs to the nearest health facility that offered HIV testing to pregnant women. Most (92%) reported that transport cost to these facilities was affordable.

Overall, nine women were not counselled for HIV testing during the just ended pregnancy. The women with known HIV status were more likely to have received counselling about HIV testing from the facilities where they attended ANC compared to those who had unknown HIV status (OR= 0.04, 95%CI=0.01 – 0.2).

The association between HIV counselling and the level of health facility where mother attended ANC was determined. Women who attended ANC in hospitals or health centre IV were 9.5 times likely to have received HIV counselling than those who attended in private clinics (OR=9.5, p value 0.003). Women who attended ANC in lower level government health centres were 2.5 times more likely to have received HIV counselling than those who attended in private clinics but this was not statistically significant (OR=2.5, p value 0.343). It was not possible to determine whether receiving HIV counselling during ANC was an effect modifier for having unknown HIV status because of the few numbers with unknown HIV status.

Among women who tested, only 3.4 % paid for the test and the median cost of the test 4000 (1.5 US$) with the inter quartile range of 2000 to 5000 Ugandan Shillings (0.7 to 1.7US$). Among women who were not tested for HIV, no mother was referred to a higher facility for HIV testing. Table 4 summarises bivariate analysis for service delivery factors

**Table 4:** Bivariate analysis for service delivery factors

	Unknown HIV status(n=10)	Known HIV Status (n=372)	Total n=382	p value
**Is there a health facility offering HIV testing in** **the woman's locality?**	**n (%)**	**n (%)**	**n (%)**	
Yes	9 (2.5)	356 (97.5)	365 (100)	0.352
**Distance to health facility (Km)**	**Median (IQR)**	**Median (IQR)**	**Median (IQR)**	
	0.5 (0.5 –1.0)	0.5 (0.25 – 1.0 )	0.5 (0.25 –1.0)	0.866
**Cost of Transport to nearest Health Facility**	**Median(IQR)**	**Median(IQR)**	**Median(IQR)**	
	0 (0 – 3000)	1000 (0 –2000)	0(0–2000)	0.986
**Received counselling for HIV testing?**	**n (%)**	**n (%)**	**n (%)**	
Yes	7 (1.9)	364 (98.1)	371 (100)	0.001
**Was the HIV test paid for?**	**n (%)**	**n (%)**	**n (%)**	
Yes	0 (0.0)	15 (100)	15 (100)	0.999
**Cost of HIV test (UGx)**	**Median (IQR)**	**Median (IQR)**	**Median (IQR)**	
	0	4000(2000–5000)	4000(2000–5000)	−--

### Knowledge on PMTCT

Almost all the women (99%) reported that it is important to know one's HIV status when pregnant. The difference in the knowledge between groups was not statistically significant. The majority (91% with known and 90% with unknown status) knew that MTCT can be prevented by taking special drugs during pregnancy. Bivariate analysis for knowledge on PMTCT has been presented in table 5. Factors with p value less than 2.0 were not included for logistic regression because they were not considered relevant as individual predictors to unknown HIV status.

**Table 5:** Bivariate analysis for knowledge on PMTCT

	Unknown HIV status(N=10)	Known HIV status(N=372)	Total(N=382)	*p* value
Does the mother think it is important to know her HIV status during pregnancy?	**n (%)**	**n (%)**	**n (%)**	
**Yes**	10 (2.6)	371 (97.4)	381 (100)	0.974

Mother's knowledge on when HIV can be transferred to her child	**n (%)**	**n (%)**	**n (%)**	

**During pregnancy**	9 (3.5)	249 (96.5)	258 (100)	0.121
**During labour**	9 (2.7)	321 (97.3)	330 (100)	0.622
**During breastfeeding**	9 (2.8)	312 (97.2)	321 (100)	0.531
**Other ways**	0 (0.0)	9 (100)	9 (100)	

Knowledge on what canbe done to reduce the risk of HIV transmission to a child during pregnancy	**n (%)**	**n (%)**	**n (%)**	
**By taking medicine during** **pregnancy**	9 (2.6)	338 (97.4)	347 (100)	0.601
**By having protected sex (condom** **use)**	8 (3.9)	197 (96.1)	205 (100)	0.085

### Multivariate analysis

Table 6 shows a summary of factors associated with unknown HIV status after multivariate analysis. We adjusted for marital status, level of health facility, male partner support and whether a participant received HIV counselling or not.

**Table 6:** Multivariate analysis for factors associated with unknown HIV status at labor

Factors	Unadjusted		Adjusted	
	Odds Ratio (95% CI)	P value	Odds Ratio (95% CI)	P value
**Marital status**				
Unmarried	1		1	
Married	0.3 (0.1 – 1.0)	0.043	0.3(0.1 – 1.4)	0.126
**Level of health centre where** **mother attended ANC**				
Private clinic	1		1	
Health centre II–III	0.3 (0.1 – 1.7)	0.172	0.3 (0.0 – 2.3)	0.249
Health centre IV / Hospital (government/NGO)	0.06 (0.01 – 0.3)	<0.001	0.09(0.01– 0.4)	0.002
**Male partner Support**				
Partner did not attend ANC	1		1	
Attended ANC with partner	0.2 (0.0– 1.8)	0.155	0.2 (0.0 – 2.4)	0.219
**Received counselling for** **HIV testing?**				
No	1		1	
Yes	0.04 (0.01 – 0.2)	<0.001	0.1 (0.01 – 0.5)	0.005

Married women were 70% less likely to present with unknown HIV status at delivery compared to the unmarried women. However, this was not statistically significant after adjusting for confounders (p value = 0.126). Partner attendance in ANC was also not statistically significant. Women who went to a higher level facility (health centre IV or government/NGO hospital) for ANC were 90% less likely to present with unknown HIV status at delivery compared to those who went to private clinics (adjusted p=0.005). There was no significant difference between those who went to private clinics and those who went to low health facility levels. Women who were offered counselling before testing for HIV were about 90% less likely to present with unknown HIV status compared to those who were not counselled (adjusted p =0.005).

## Discussion

The above results show that uptake of HIV testing during ANC among women delivering in Mulago Hospital is almost 100%. Nevertheless, a few women (2.6%) presented in labour with unknown HIV status. The prevalence of unknown HIV status at labour increased from 2.6% to 7.9% when participants who reported in labour without evidence of testing for HIV are considered as having unknown HIV status, a criterion that was used by Ononge etal^13^ in 2006. In practice, evidence of HIV testing is critical to determine intervention to prevent mother to child transmission of HIV. However, lack of documentation by health workers, loss of documents and forgetting to bring documents to hospital in view of the unpredictable timing of labour can cause women to report without evidence of HIV testing. Indeed, studies have reported knowledge of HIV status to be the commonest reason for refusal to test for HIV at labour and delivery despite this being undocumented^15^,^16^.

This prevalence of unknown HIV status is far much below the Uganda National average of 40% of women who do not test during pregnancy^17^ and other Ugandan population based studies (40%)^12^. This discrepancy could be due to the fact that this was a hospital- based study. However, it is also significantly less than that reported in other hospital- based studies in sub-Saharan Africa like Nigeria (34%) and the previous study in Mulago Hospital (27.1%) before PICT was rolled out in Uganda^13^,^18^. If documentation of HIV status is considered as a defining criterion, the prevalence of 7.9% is almost similar to other hospital- based studies in Botswana and South Africa with prevalence rates ranging from 9% to 15%^16^,^19^, ^20^. The prevalence in these studies could probably be much lower if only self-report was considered as having unknown HIV status.

The independent factors associated with unknown HIV status were level of health facility where mother attended ANC and whether mother received counselling for HIV testing or not. Women who attended ANC in hospitals or health centre IVs were 90% less likely to have unknown HIV status at labour and nine times more likely to have been counselled for HIV than those who attended ANC in Health Centre III and II and in private clinics. This finding is also consistent with the previous study in Mulago Hospital and another population study in Eastern Uganda^12^. Furthermore, those who received HIV counselling in ANC were 90% less likely to present with unknown HIV status at labour than those who did not. These results may be a hint that HIV counselling in private clinics and lower Health centres may not be routine as stipulated in the Ugandan HIV testing policy. The fact that these women were not referred elsewhere for testing reflects a gap in their ANC services. The higher level facilities were more likely to offer HIV counselling probably because they may have higher staff numbers to handle the work load, better trained staff, more government resources and greater exposure to Ministry of Health guidelines than the lower level or private facilities.

Marital status and male partner support were not associated with unknown HIV status probably due to the small number of women with unknown HIV status.

Studies in sub-Saharan Africa have indicated that lack of infrastructure and adequate health personnel to conduct HCT, stigma and discrimination, attitudes and skills of health care workers have been sighted as setbacks in the provision of HCT to pregnant women^21^. In this study, based on the reasons given for not testing, these factors did not play a major role especially in public/government accredited health facilities; however, apart from lack of counselling and to a very small degree the cost of the test, it is not known to what extent these factors affected provision of HCT services in private clinics. To improve HCT services in private clinics, the government could consider supporting the provision of quality and free HCT services in private clinics as well as urge them to refer women if they cannot offer HIV testing.

Fifty percent (5/10) of the women who had unknown HIV status mentioned “personal reasons” as to why they “opted out” of HCT during ANC. This shows that HIV testing in developing countries follows an “opt out” approach and some women exercise their right to refuse the test contrary to previous studies that report that women are unaware of their choice to opt out^22^. However, this could compliment the argument that HIV testing should be mandatory in ANC in order to protect the unborn child^23^.

In contrast to the other studies^13^,^24^, multi-parity, age, education level, socio-economic status, gestational age at first ANC and knowledge of PMTCT were not associated with having unknown HIV status in labour. Perhaps, bringing PMTCT services closer to the population could have negated the effect of some of these factors on unknown HIV status in labour, especially education level and socio-economic status as has been argued by Larson^12^.

All women with unknown HIV status eventually tested at labor ward, although 80% were tested after delivery. This shows that there is chance that a woman will miss testing before delivery and if HIV infected will miss the minimum maternal intervention at labor for PMTCT due to obstetric emergencies as has already been observed in other studies^25^. The uptake of HIV testing at the labor ward was 100% probably because the need for a decision whether or not to provide PMTCT interventions outweighed other personal factors at that time.

The limitation of this study was that it was hospital-based and mainly depended on self-reports and this could have caused reporting bias. In addition, women delivering at a tertiary hospital may be different from those that deliver at lower level facilities because of the mechanisms involved in a referral system. Results of this study may therefore not be generalizable.

We recommend that the government supports private clinics and lower level facilities to provide quality HCT services in their antenatal clinics and to refer to higher facilities for HIV testing if this is not offered.
